# Stroke awareness in the general population: knowledge of stroke risk factors and warning signs in older adults

**DOI:** 10.1186/1471-2318-9-35

**Published:** 2009-08-05

**Authors:** Anne Hickey, Ann O'Hanlon, Hannah McGee, Claire Donnellan, Emer Shelley, Frances Horgan, Desmond O'Neill

**Affiliations:** 1 Department of Psychology, Division of Population Health Sciences, Royal College of Surgeons in Ireland, Dublin, Ireland; 2 Department of Medical Gerontology, Trinity College, Dublin, Ireland; 3 Department of Epidemiology and Public Health Medicine, Division of Population Health Sciences, Royal College of Surgeons in Ireland, Dublin, Ireland; 4 School of Physiotherapy, Royal College of Surgeons in Ireland, Dublin, Ireland

## Abstract

**Background:**

Stroke is a leading cause of death and functional impairment. While older people are particularly vulnerable to stroke, research suggests that they have the poorest awareness of stroke warning signs and risk factors. This study examined knowledge of stroke warning signs and risk factors among community-dwelling older adults.

**Methods:**

Randomly selected community-dwelling older people (aged 65+) in Ireland (n = 2,033; 68% response rate). Participants completed home interviews. Questions assessed knowledge of stroke warning signs and risk factors, and personal risk factors for stroke.

**Results:**

Of the overall sample, 6% had previously experienced a stroke or transient ischaemic attack. When asked to identify stroke risk factors from a provided list, less than half of the overall sample identified established risk factors (e.g., smoking, hypercholesterolaemia), hypertension being the only exception (identified by 74%). Similarly, less than half identified established warning signs (e.g., weakness, headache), with slurred speech (54%) as the exception. Overall, there were considerable gaps in awareness with poorest levels evident in those with primary level education only and in those living in Northern Ireland (compared with Republic of Ireland).

**Conclusion:**

Knowledge deficits in this study suggest that most of the common early symptoms or signs of stroke were recognized as such by less than half of the older adults surveyed. As such, many older adults may not recognise early symptoms of stroke in themselves or others. Thus, they may lose vital time in presenting for medical attention. Lack of public awareness about stroke warning signs and risk factors must be addressed as one important contribution to reducing mortality and morbidity from stroke.

## Background

Speedy access to acute medical services, particularly thrombolysis, is an important predictor of stroke outcome [1-4]. Rapid access to services requires that there is understanding of the warning signs for stroke. Knowledge of risk factors and warning signs in the general population has consistently been found to be poor, with knowledge levels poorest in groups that have the highest risk of stroke, e.g., those aged over 75 [5-10]. Even among those aware that they have a risk factor for stroke, knowledge of stroke warning signs has been found to be no better than for those without risk factors [11].

Of all neurological diseases, stroke is the most preventable. Many of the established risk factors for stroke, including hypertension, high cholesterol, diabetes, heart disease and smoking can be prevented either through more healthy lifestyle choices or by medication. Greater understanding of perceived risk factors and warning signs for stroke would facilitate health interventions aimed at reducing morbidity and mortality from stroke. These interventions do not need to be complex. For instance, provision of an information booklet on stroke significantly increased the knowledge of risk factors and warning signs in a sample of hospitalised stroke patients and their carers [12]. The need to increase public awareness of stroke risk factors and warning signs has been identified as critical to addressing the large gaps in knowledge [13,14], including promoting awareness about the seriousness of stroke, and the value of speedy evaluation to maximise therapeutic outcomes [8,14]. Given that older people are the most vulnerable demographic grouping at risk for stroke, it is particularly important that the factors associated with low awareness are understood.

Population-based studies demonstrate sub-optimal awareness of stroke risk factors and warning signs for stroke onset [5,7,8,10,15,16]. Similar findings are evident in patients interviewed following hospital admission for stroke [17,18]. Contemporary European data on stroke awareness in the general population is scarce [19]. Comparisons across countries could inform about common knowledge deficits and about effective strategies for improving awareness for, and response to, a condition where early identification is so crucial – i.e., where 'time is brain'. Study findings to date indicate a significant improvement in population awareness of stroke warning signs following public education campaigns [8,10,20], although frequently less effective in older people [10,16]. This increase in knowledge appears also to have a significant effect on presentations to Accident and Emergency departments [21]. There appear to be differences in the efficacy of campaigns depending on the media strategy used [16]. However, the impact of media campaigns on knowledge levels has been shown to decline significantly once the media campaign finishes [21].

The present study was included as part of a large population survey of randomly selected community-dwelling older Irish adults conducted across the island of Ireland (Republic and Northern Ireland) [22]. Levels of community awareness of stroke warning signs and risk factors has not been established in the Irish context and this study provided the opportunity to compare representative population samples in two neighbouring jurisdictions with different health systems. While public education strategies about stroke did not differ across the two regions at the time of the survey, service structures differ – for instance there are many more stroke units in hospitals in Northern Ireland [23], with consequent better access to post-stroke rehabilitation. This study thus sought to identify levels of knowledge and correlates of stroke warning signs and risk factors in community-dwelling older adults, a group whose age places them at higher risk for stroke. In addition, it compared levels of stroke risk awareness across two jurisdictions – the Republic of Ireland and Northern Ireland.

## Methods

### Sample and procedure

This study involved a cross-sectional survey of randomly selected community-based older people (aged 65+) in the Republic of Ireland and Northern Ireland. The survey was conducted as part of the Healthy Ageing Research Programme (HARP), a cross-border research programme funded by the Irish Health Research Board (HRB). The aim of HARP was to examine the health and social service use of older people in both jurisdictions, and to study how health and health systems enable or impede successful ageing. The survey was broad-based, including questions on functional ability, psychological well-being, quality of life, health-care and social service use, and quality of health-care [22]. The questions on which this study is based formed a small sub-set of questions in the overall survey instrument. In the pilot version of the questionnaire, questions addressing risk factors and warning signs for stroke were open-ended, based on the questions used by Schneider and colleagues [8]. However, in piloting the survey instrument, these questions were found to require too much time in the context of the overall survey, and it was decided that a list format would be used, with respondents identifying from a list those factors that were considered risk factors and warning signs for stroke (one list provided for each).

Survey participants were identified by the most complete population listings (Register of Electors and postal address files in the Republic of Ireland and Northern Ireland, respectively). Participants were selected using a computer-based random sampling system. Interviews were conducted in participants' own homes by trained market researchers from a market research agency based in Dublin and Belfast in the Republic of Ireland and Northern Ireland, respectively. The overall response rate in the Republic of Ireland was 64% and in Northern Ireland was 89%. The sample was broadly representative of the older population profile in both jurisdictions in Ireland [24,25].

### Measures

#### Demographic variables

Age, gender, education, marital status, residential location (urban/rural) and geographic location (Republic of Ireland/Northern Ireland).

#### Knowledge of stroke warning signs and risk factors

Participants were first asked if they had had a stroke. They were then asked to identify stroke warning signs and risk factors. Warning signs and risk factors were shown to participants in a list format, derived from Schneider *et al*.'s US survey [8]. Based on warning signs established by the American Stroke Association, National Stroke Association and the National Institute of Neurological Disorders and Stroke, the following were listed as important warning signs of stroke: sudden numbness or weakness in the face, arm or leg, especially on one side of the body; sudden confusion or difficulty speaking or understanding speech; sudden trouble seeing in one or both eyes; sudden difficulty in walking, dizziness or loss of balance/coordination; or sudden and severe headache with no known cause. A list of established stroke risk factors was also derived [8]. These risk factors included hypertension, smoking, hypercholesterolemia, heart disease, diabetes, heavy alcohol use and transient ischaemic attack (TIA) or prior stroke.

Schneider and colleagues [8] used open-ended questions when asking about stroke risk factors and warning signs. However, pilot work with the original open-ended questions showed this approach to be too time-consuming in the context of a very broad-based health and healthcare survey. Therefore, the protocol was amended to ask respondents to identify key stroke warning signs and risk factors, if any, from a provided list. This approach potentially simplified the task by transforming it from primarily a memory to a recognition task. This was taken into consideration when analysing study findings. Thus, rather than examining the correlates of correct identification of warning signs and risk factors based on getting one or more of these correct, as in the Schneider et al. study, the number of correct responses was set at a minimum of two, in order to compensate for this comparatively less difficult recognition task.

#### Personal risk factors

Associations between personal risk factors and knowledge of stroke warning signs and risk factors were also examined. Personal risk factors included having heart disease (including hypertension), being a current or past smoker, having a prior stroke and taking regular exercise.

### Analyses

As is standard with population survey data, the data collected was statistically adjusted or "re-weighted" prior to analysis. This re-weighting adjusts the results to compensate for the over-representation or under-representation of particular population subgroups in the sample, introducing potential bias which may arise from issues related to sample design and also to differential non-response within sub-groups of the population. The re-weighting procedure used was based on a minimum information loss algorithm, which adjusts an initial weight so as to ensure that the distributional characteristics of the sample matches those of the population according to independent national sources. In the case of the Republic of Ireland and Northern Ireland, data was re-weighted based on the most recent Census information for that region [24,25]. The variables used in the statistical adjustment procedure were sex, age group, and residential category (urban/rural). Results could thus be considered as broadly representative of the general population of older people in Ireland.

Descriptive and comparative analyses were carried out using Stata Version 8.2. The chi-square test was used to examine differences between the Republic of Ireland and Northern Ireland in recognition of stroke risk factors and warning signs. Logistic regression analysis was used to examine the effects of demographic variables and the presence of risk factors on stroke knowledge; variables included in this analysis included age; gender; education (completed primary education or less (60% of sample overall) versus completed second level education or more); home and geographic location (Republic versus Northern Ireland); residential location (urban versus rural; rural defined as living in the countryside, or in a town or village with a population of less than 1,500 people); and self-reported risk factors, such as hypertension, prior stroke, and cigarette smoking.

### Ethics

Ethics approval was granted by the Research Ethics Committee of the Royal College of Surgeons in Ireland.

## Results

Overall, there was a 68% participation rate. The overall sample (n = 2,033) was broadly representative of the older population profile in Ireland [22]. The total sample is profiled in Table 1 alongside the results for the Republic of Ireland and Northern Ireland samples. While both samples were selected randomly from neighbouring jurisdictions, there were significant demographic differences between the samples, the Northern Ireland sample being older, less educated, and with a poorer cardiovascular profile. Six per cent of respondents had a previous stroke, twice as many in Northern Ireland as in the Republic of Ireland (8% vs 4%).

**Table 1:** Comparisons of demographic profiles and prevalence of cardiovascular risk factors between the community-dwelling older population in Northern Ireland and the Republic of Ireland*.

	Overall sample (n = 2, 033)	Republic of Ireland (n = 1, 033)	Northern Ireland (n = 1,000)	Difference between regions(χ^2^)
Age, mean (SD) (range)	74.1 (6.8) (65–102)	74.3 (6.8) (65–102)	75.3 (7.0) (65–99)	p < 0.01
Gender: Women, % (N)	57% (1,171)	56% (578)	58% (580)	p = 0.38, NS
Education (> 14 years full-time), % (N)	36% (748)	42% (440)	31% (308)	p < 0.0001
Self-reported risk factors: % (N)				
- past smoker	36% (729)	41% (432)	30% (297)	p < 0.0001
- current smoker	17% (358)	17% (178)	18% (180)	p = 0.49, NS
- history of heart disease	25% (508)	21% (216)	29% (292)	p < 0.0001
- prior stroke	6% (121)	4% (45)	8% (76)	p < 0.01

*Weighted data

### Knowledge of stroke warning signs

Knowledge of stroke warning signs and risk factors in the overall population and in the two jurisdictions is presented in Table 2. Warning signs identified by at least 5% of the study sample are shown. The warning signs most commonly identified were slurred speech, dizziness, numbness, weakness and headache. However, with the exception of slurred speech (identified by 54%), less than half of the population identified these established warning signs. Significantly more of the Republic of Ireland than the Northern Ireland sample identified the established warning signs of dizziness, numbness, problems with vision and difficulty understanding. Significantly more of the Northern Ireland than the Republic of Ireland sample identified weakness as a warning sign for stroke. A significantly greater proportion in Northern Ireland than in the Republic (18% vs 7%) identified no warning signs for stroke.

**Table 2:** Perception of stroke warning signs and risk factors in the community-dwelling older population in Northern Ireland and the Republic of Ireland**.

Responses	Overall sample (n = 2, 033) %(N)	Republic of Ireland (n = 1,033) %(N)	Northern Ireland (n = 1,000) %(N)	Difference between regions (χ^2^)
Warning signs				
- Slurred speech *	54% (1,102)	54% (569)	53% (533)	p = 0.93, NS
- Dizziness*	44% (894)	51% (532)	36% (361)	p < 0.0001
- Numbness (any)*	41% (832)	45% (472)	36% (360)	p = 0.0001
- Weakness (any) *	38% (790)	36% (374)	41% (414)	p < 0.01
- Headache*	29% (600)	30% (316)	28% (284)	p = 0.53, NS
- Vision problems*	20% (410)	22% (230)	18% (180)	p < 0.05
- Difficulty understanding	18% (369)	22% (230)	14% (139)	p < 0.0001
No response/don't know	13% (255)	7% (74)	18% (181)	p < 0.0001
Risk factors				
- Hypertension*	75% (1,523)	75% (786)	75% (737)	p = 0.91, NS
- Stress	43% (888)	43% (450)	44% (436)	p = 0.6, NS
- Cholesterol*	40% (823)	45% (471)	35% (353)	p < 0.0001
- Smoking*	30% (621)	29% (300)	32% (320)	p = 0.07, NS
- Obesity	30% (608)	26% (273)	33% (334)	p = 0.0001
- Lack of exercise	18% (363)	19% (198)	17% (165)	p = 0.22, NS
- Family history of stroke	16% (333)	22% (227)	10% (103)	p < 0.0001
- Diabetes*	11% (217)	12% (124)	9% (92)	p = 0.08, NS
- Alcohol use*	10% (201)	9% (93)	11% (108)	p = 0.14, NS
- No response/don't know	6% (124)	4% (48)	8% (76)	p < 0.01

Note: Only factors where at least 5% indicated relevance are listed. Data represents up to 3 responses per participants and those include responses that have not been established by the medical community as stroke warning signs or stroke risk factors

* Established warning sign/risk factor

** Weighted data

The association between demographic factors and identification of stroke warning signs (defined as correct identification of 2 or more warning signs) is presented in Table S1 (see additional file 1). Adjusted odds ratio analysis indicates that higher levels of knowledge were significantly associated with having second level education or greater (OR = 1.9, p < 0.001) and geographic location (living in the Republic of Ireland) (OR = 2.1, p < 0.001).

### Knowledge of stroke risk factors

Risk factors identified by at least 5% of both study samples are shown in Table 2. The most commonly identified stroke risk factors by the overall sample were hypertension, stress, hypercholesterolaemia, smoking and obesity. However, with the exception of hypertension (identified by 74%), less than half of the population correctly identified established stroke risk factors. A number of significant differences in risk factor recognition were found between the Republic of Ireland and Northern Ireland samples. Those resident in the Republic were significantly more likely to identify hypercholesterolaemia and family history as important risk factors, while those resident in Northern Ireland were significantly more likely to identify obesity as a risk factor. The percentage unable to identify any stroke risk factors was small but twice the level in Northern Ireland compared to the Republic of Ireland (8% vs 4%, p < .01).

Analysis of the association between demographic factors and identification of correct stroke risk factors (defined as identification of 2 or more accurate risk factors) is presented in Table S1 (see additional file 1). No significant associations were found.

The association between presence of risk factors and correct identification of stroke warning signs and risk factors was examined (Table S2 (additional file 1)). Having heart disease or a prior stroke or TIA was not associated with higher knowledge of stroke warning signs. Current smokers and those reporting not engaging in regular exercise had significantly lower levels of knowledge about stroke warning signs (OR = 0.62, p < 0.01; OR = 0.65, p < 0.01, respectively).

In terms of identification of stroke risk factors, participants with a specific self-reported risk factor were no more likely than others to correctly identify stroke risk factors (see Table S2 (additional file 1)).

Finally, the relationship between having a risk factor and identifying that factor as a risk factor for stroke was examined. A significant relationship was found for smoking, with current and past smokers significantly more likely to identify smoking as a stroke risk factor than never smokers (OR = 1.8, p < 0.0001; OR = 1.4, p < 0.005, respectively).

## Discussion

This paper reports knowledge of stroke risk factors and warning signs for stroke in national community samples of older adults in two neighbouring jurisdictions.

Consistent with the findings of other studies, this survey found that knowledge of stroke warning signs was poor. When presented with a list of warning signs, only one (slurred speech) was identified by more than half of respondents. This finding confirms previous studies, in which dizziness and numbness were identified [5,8], and contrasts somewhat with other study findings, where disturbance of vision was found to be the most commonly identified warning sign [11]. Notably, one in ten could identify no warning signs despite being presented with a list.

Hypertension was identified most frequently as a risk factor for stroke, followed by stress, hypercholesterolaemia, smoking and obesity, findings similar to those reported by Pancioli and colleagues [5]. However, while hypertension was identified as a stroke risk factor by three-quarters of the respondents in this survey, all other risk factors were identified by less than half with 6% of the sample unable to identify any risk factor. Thus, while this Irish population had greater awareness of stroke risk factors than a younger US sample [8], albeit in a recognition-type task of identifying factors from a list, there were still considerable gaps in awareness. In addition, factors such as stress and obesity were commonly identified, although they are not established as risk factors for stroke. In contrast, established risk factors such as diabetes and alcohol use were identified by approximately one in ten respondents. Health promotion in this area could provide clarification of the similarities in risk factors for stroke and MI, with more specific information on factors that increase risk specifically for stroke.

The poorer level of awareness of stroke warning signs relative to risk factors is consistent with previous reports [7,10,11,14,19] and is cause for concern, given that most of the common early symptoms or signs of stroke were recognised as such by less than half of the older adults surveyed. As such, many older adults in this study may not recognise that they, or a significant other, are having a stroke when symptoms emerge, thus losing vital time in presenting for medical attention. Mass media campaigns to improve public awareness of stroke warning signs have been found to be effective in improving knowledge of warning signs [8,10,16,20,26], particularly in younger age groups, although producing little change in knowledge of risk factors [10]. However, in many studies, these campaigns have been found to be less effective for those aged 65+ [10,16], although this is not a universal finding [20]. People in younger age groups have been shown to be more knowledgeable than older people prior to intervention with public health promotion campaigns and to remain more knowledgeable after the campaign [16]. However, there is evidence that television based advertising may contribute to a reduction in age-related differences in knowledge of stroke warning signs [21]. Older people – who are at greater risk for stroke because of their age – are a particularly important population sub-group to target in relation to awareness of stroke warning signs. To date, evidence indicates that stroke awareness campaigns are least effective in increasing knowledge in this older age group. A lack of public awareness in relation to these factors will translate into failure to reduce mortality and morbidity from stroke over time [14]. In addition, research evidence indicates that increasing public awareness of stroke warning signs does not translate necessarily to improving timely access to medical care [20,27]. Almost 40% of this study sample lived in rural areas [22]. The inability to identify and respond to stroke warning signs in a rural context, where distance from hospital is an added obstacle to accessing rapid medical care, highlights an area of specific need for health promotion intervention.

Limitations of this study include that two different market research companies gathered data in two different jurisdictions, yielding a different response rate in each jurisdiction. It is possible that there was variation between interviewers. However, the research team sought to minimize this possibility by having all interviewers receive the same training and work from a standardised script. The issue of non-response bias was greater in the Republic of Ireland, where the response rate was lower (64%) than in Northern Ireland (89%). The use of a list format in identifying stroke risk factors and warning signs may have resulted in an over-estimate of knowledge of stroke risk factors and warning signs than if open-ended questions had been used. There is evidence that respondents are better at recognition of risk factors and warning signs than they are at identifying them in response to an open-ended ("unaided") question [19,20]. Research evidence about stroke knowledge in older populations in other countries is relatively sparse and so comparisons with this study are limited. Also limited is evidence from this survey of the benefits of knowledge, such as impact on recognition and action concerning signs and symptoms of stroke or on changes in risk behaviours (e.g., adopting more healthy lifestyle choices). Such questions cannot be addressed by cross-sectional studies such as this and need more longitudinal study investment.

Reasons for differences in the two jurisdictions are unclear. Neither had a concerted campaign promoting knowledge of stroke in the community at the time of the survey. However, since both groups surveyed have poor records regarding stroke awareness, there is little to be gained from determining why one fared worse than the other. Public campaigns are clearly needed in both jurisdictions. A new campaign (FAST) has been launched by the National Health Service in the UK system, including Northern Ireland. Plans are underway to provide a similar programme in the Republic of Ireland. This survey thus provides a useful baseline for these programmes.

## Conclusion

Early recognition of stroke symptoms and signs is key to maximising the potential for medical intervention and more favourable stroke outcomes – the 'time is brain' imperative. This study highlights significant gaps in awareness in relation to stroke warning signs and risk factors in older people in two jurisdictions. They are unlikely to be alone – assessment of stroke awareness at general population level is needed more widely if we are to improve stroke care across Europe. The need for substantial population health education with regard to stroke prevention and management is critical to a future reduction in both the incidence of stroke and in reduction of stroke mortality and morbidity [28]. This will require a more concerted effort across specialists in stroke care, public health and geriatric medicine to ensure that programmes developed to meet this need are based on sound gerontological and public health principles [29,30].

## Competing interests

The authors declare that they have no competing interests.

## Authors' contributions

AH was the lead investigator for this sub-study of the overall general population study. She identified and piloted questions, guided analysis, and was lead writer of this manuscript. AO'H was the overall project co-ordinator and was involved in study planning, analysis of study findings, and contributed to preparation of this manuscript. HMcG was the overall population study principal investigator, was involved in study planning and planning of analysis, and contributed to preparation of this manuscript. CD was a member of the study research team and was involved in analysis of the study findings and contributed to preparation of this manuscript. ES was a member of the national research project steering committee and was involved in study planning and contributed to preparation of this manuscript. FH was a member of the national research project steering committee and was involved in study planning and contributed to preparation of this manuscript. DO'N was an overall population study co-investigator and was involved in study planning and contributed to preparation of this manuscript. All authors read and approved the final manuscript.

## Pre-publication history

The pre-publication history for this paper can be accessed here:



## Supplementary Material

Additional file 1 **Supplemental Tables**. *Table S1*: Factors associated with knowledge of stroke warning signs and risk factors (minimum 2 items correct in each case). *Table S2*: Relationship between presence of personal risk factors and knowledge of stroke warning signs and risk factors (minimum 2 items correct in each case) Click here for file
