# Supplementary material for: Stroke awareness in the general population: knowledge of stroke risk factors and warning signs in older adults
Source: BMC Geriatr. 2009 Aug 5;9:35. doi: 10.1186/1471-2318-9-35 (PMC2734750; doi:10.1186/1471-2318-9-35)
Supplement: Additional file 1 — Supplemental Tables. Table S1: Factors associated with knowledge of stroke warning signs and risk factors (minimum 2 items correct in each case). Table S2: Relationship between presence of personal risk factors and knowledge of stroke warning signs and risk factors (minimum 2 items correct in each case) [file 1471-2318-9-35-S1.doc]

**Table S1.** Factors associated with knowledge of stroke warning signs and risk factors

(minimum 2 items correct in each case)

| Socio-demographic variables | Warning signs | | | | Risk factors | | | |
| --- | --- | --- | --- | --- | --- | --- | --- | --- |
|  | Number correct | | Adjusted  odds ratio | 95%  C.I. | Number correct | | Adjusted  odds ratio | 95%  C.I. |
|  | <2  % (n) | >2  % (n) |  |  | <2  % (n) | >2  % (n) |  |  |
| Gender:  Men  Women | 15 (124)  14 (164) | 85 (753)  86 (992) | 1.1 | 0.83-1.53 | 37 (328)  37 (429) | 63 (549)  63 (727) | 0.99 | 0.8-1.23 |
| Age  ≤ 74 years  75+ years | 13 (141)  16 (144) | 87 (1,021)  84 (716) | 0.86 | 0.64-1.15 | 35 (409)  39 (342) | 65 (753)  61 (518) | 0.85 | 0.7-1.05 |
| Education:  Primary or less  Second level+ | 17 (209)  10 (77) | 83 (1,024)  90 (708) | 1.7 | 1.2-2.4 | 37 (468)  36 (283) | 63 (765)  64 (499) | 1.0 | 0.84-1.27 |
| Marital status:  Married  Single  Sep/divorced  Widowed | 16 (33)  12 (103)  11 (9)  17 (136) | 84 (190)  88 (748)  89 (77)  83 (663) | Reference  1.0  1.6  0.80 | 0.57-1.7  0.66-3.9  0.49-1.34 | 37 (82)  37 (317)  32 (27)  36 (302) | 63 (141)  63 (534)  68 (59)  64 (497) | Reference  1.0  1.1  1.0 | 0.67-1.4  0.66-2.0  0.72-1.4 |
| Living status:  Lives with others  Lives alone | 12 (138)  17 (148) | 88 (1,005)  83 (727) | 0.92 | 0.58-1.4 | 37 (432)  36 (317) | 63 (711)  64 (558) | 1.1 | 0.82-1.48 |
| Residential location:  Urban^1^  Rural | 13 (173)  16 (111) | 87 (1,083)  84 (634) | 1.0 | 0.98-1.01 | 35 (444)  40 (298) | 65 (812)  60 (447) | 0.99 | 0.98-1.0 |
| Geographical location:  N. Ireland  RoI | 19 (191) 10 (97) | 81 (796)  90 (949) | 2.1 | 1.53-2.9 | 36 (372)  37 (385) | 64 (615)  63 (661) | 1.0 | 0.84-1.31 |

Note ^1^defined as towns of 1,500+ people. Adjusted odds ratio controls for all socio-demographic variables

**Table S2.** Relationship between presence of personal risk factors and knowledge of stroke warning signs and risk factors

**(minimum 2 items correct in each case)**

|  | Warning signs | | | | Risk factors | | | |
| --- | --- | --- | --- | --- | --- | --- | --- | --- |
| Self-reported risk factors | Number correct | | Adjusted  odds ratio | 95%  C.I. | Number correct | | Adjusted  odds ratio | 95%  C.I. |
|  | <2  % (n) | >2  % (n) |  |  | <2  % (n) | >2  % (n) |  |  |
| - heart disease  No  Yes | 14 (212)  14 (70) | 86 (1,295)  86 (438) | 1.0 | 0.74-1.4 | 37 (556)  37 (193) | 63 (951)  63 (315) | 0.98 | 0.78-1.24 |
| - past smoker  No  Yes | 13 (133)  13 (88) | 87 (817)  87 (641) | 1.0 | 0.75-1.4 | 39 (367)  37 (269) | 61 (583)  63 (460) | 1.0 | 0.88-1.4 |
| - current smoker  No  Yes | 13 (221)  20 (67) | 87 (1,458)  80 (287) | 0.62 | 0.45-0.87 | 38 (636)  32 (121) | 62 (1,043)  68 (233) | 1.3 | 1.0-1.6 |
| - prior stroke  No  Yes | 12 (185)  13 (17 | 88 (1,309)  87 (104) | 1.1 | 0.63-2.0 | 36 (549)  43 (51) | 63 (945)  57 (70) | 0.75 | 0.5-1.1 |
| - exercise enough  Yes  No | 12 (153)  19 (134) | 88 (1,187)  81 (546) | 0.65 | 0.5-0.87 | 37 (488)  37 (262) | 63 (852)  63 (418) | 0.99 | 0.8-1.2 |

Note: Adjusted odds ratio controls in warning signs controls for education and geographical location
